# Artistic Skills Recovery and Compensation in Visual Artists after Stroke

**DOI:** 10.3389/fneur.2016.00076

**Published:** 2016-05-13

**Authors:** Eugen Bogdan Petcu, Katherine Sherwood, Aurel Popa-Wagner, Ana Maria Buga, Lanfranco Aceti, Rodica Ileana Miroiu

**Affiliations:** ^1^Griffith University School of Medicine, Gold Coast Campus, QLD, USA; ^2^Queensland Eye Institute, Brisbane, QLD, Australia; ^3^Art Department and Disability Studies Program, University of California Berkeley, Berkeley, CA, USA; ^4^Department of Psychiatry, University of Medicine Rostock, Rostock, Germany; ^5^Center of Clinical and Experimental Research, University of Medicine and Pharmacy Craiova, Craiova, Romania; ^6^Arts Administration, Boston University, Boston, MA, USA; ^7^Griffith University School of Dentistry and Oral Health, Gold Coast Campus, QLD, Australia

**Keywords:** artistic skills recovery, compensation, functional magnetic resonance imaging, stroke, prefrontal lobe, parietal lobe, molecular

## Abstract

**Background:**

Art is a characteristic of mankind, which requires superior central nervous processing and integration of motor functions with visual information. At the present time, a significant amount of information related to neurobiological basis of artistic creation has been derived from neuro-radiological cognitive studies, which have revealed that subsequent to tissue destruction, the artists continue to create art. The current study aims to review the most important cases of visual artists with stroke and to discuss artistic skills recovery and compensation as well as artistic style after stroke.

**Methods:**

The role of various central nervous system regions in artistic creation was reviewed on the basis of previously published functional studies. Our PubMed search (1995–2015) has identified 10 famous artists with right cerebral stroke as well as 5 with left cerebral stroke who survived and continued to create art after stroke. As the artists included in this review lived at various times during the twentieth century and in different countries, clinical information related to their case was limited. However, it appears that artistic skills recovery and compensation appear within days after stroke. Some of the artists would subsequently change their artistic style. All these elements have been evaluated within the context of specific clinical cases.

**Conclusion:**

The poststroke artistic skills recovery and compensation with development of a new style or the opposite, regaining the previous prestroke style, represents a significant element of clinical importance in medical rehabilitation as well as neuroesthetics, which requires further evaluation. At the present time, the molecular mechanisms of artistic creation are poorly understood, and more standardized clinical and experimental studies are needed.

## Introduction

Visual artistic creation represents one of the most intriguing characteristics of mankind. The artists and the public are inherently linked by the actual act of art appraisal, which cannot be achieved without a superior integration of the cognitive functions. Moreover, concerning the artist, his work requires superior processing and fine tuning of fine motor skills and visual information. However, both the artists and spectators must be capable of relating to each other, at various degrees, *via* a set of complex neuro-psychological and philosophical concepts defined within and decontextualized from a series of specific social environments. Without any doubt, the artistic creation and esthetic evaluation of artistic output represents one of many facets of central nervous functionality. In this context, neuroesthetics is advocated by some authors as a “science” bordering arts, medicine, neurosciences, and empiric esthetics, capable of explaining various processes associated with neurobiology of art. Perhaps, the grandfather of neuroesthetics could be considered Giovanni Paolo Lomazzo (1538–1600) who was the first to describe the link between emotional states and colors in his “Trattato dell’arte della pittura, scultura et architettura” published in 1584 (1). However, it would have been impossible to attempt an initial description of neuroesthetics without functional magnetic resonance imaging (fMRI) and magnetoencephalography (MEG), non-invasive methods, which allow an efficient real-time functional evaluation of various brain structures. Before the advent of these revolutionary methods of investigation, the role of various parts of the brain was established after experimental and incidental trauma to the brain. The most well-known such case is the discovery of prefrontal cortex function in 1868, in a railroad worker who survived an accident, which destroyed his anterior portion of the left frontal lobe (2). Although surprisingly, the accident had no impact on the motor function, language, intellect and memory of Phineas Gage, it was soon obvious that his personality has changed dramatically. Formerly known as a gentle person, hard-working and trustworthy he changed so much that he became “no longer Gage” for his entourage displaying serious behavioral problems which were not present before the accident. Therefore, it was suggested that frontal lobe modulates personality and behavior (3). In 1994, a possible destruction of the ventral–medial region of the frontal lobe was indicated after an extensive evaluation of Gage’s skull by modern radiological methods (2). If we extrapolate the principles suggested by this case, it becomes obvious that the initial information on neurobiology of art could be derived from clinical observational cases of artists that had developed central nervous lesions associated with tissue destruction such as stroke. Theoretically, stroke is associated with arrest of the activity in some cerebral areas and centers but remarkably some of these artists–patients have shown recovery and compensation of their artistic skills. Meantime, based on functional radiological studies (fMRI, CT), other authors have reported additional information, which could explain the complex central nervous system processing associated with art evaluation and potentially with artistic creation. Although both categories of studies, clinical reports as well as prospective functional evaluations have attempted to define the basic mechanisms of art creativity and interpretation, this is far from been achieved. At the present time, it is obvious that art creation represents the result of both psychological and morphological changes associated with functional states and/or central nervous system pathology described in an individual living in a specific cultural context. However, there is an acute need for experimental standardization and new artistic-clinical parameters in order to facilitate the understanding of neurobiology of artistic creation and its appraisal. In this manuscript, we will review the current knowledge related to the role of various regions of the central nervous system in artistic creation and appraisal in parallel with selected cases of famous visual artists with stroke trying to identify the most important aspects related to poststroke artistic creation and interpretation including the recovery and compensation of the artistic skills.

### The Role of (Pre)frontal Lobe in Artistic Creation

Artistic creation represents a complex sensory–motor act modulated by the personality of artists as well as by numerous cultural, religious, and philosophical factors. It also depends on the integrity of the structures which modulate memory, attention, and semantics.

Recently, fMRI studies conducted in patients with severe abnormalities related to thought, memory, and attention, such as schizophrenia, have reported abnormalities in the prefrontal cortex/inferior frontal gyrus. It is suggested that the background of these functional issues would be related to a severe disconnection between the frontal and the parietal/temporal cortex affecting the left semantic network (4). Also in schizophrenic patients, Brodman area 9 of frontal lobe displays an altered gene expression profile in parallel with attention deficit problems, hallucinations, and delirium (5). At the present time, it is accepted that there is a strong link between the microscopic and macroscopic morphology of the (pre)frontal lobe and personality of the subjects. More specifically, extroversion is associated with an increased volume of the orbitofrontal area, which regulates reward processing. Remarkably, it seems that the lateral (pre) frontal cortex is associated not only with voluntary modulation of behavior but also with conscientiousness (6). However, the prefrontal cortex represents the most specialized structure, which mediates artistic creation and appreciation. Several groups have reported that medial orbito-frontal lobe represents the integrative center for evaluation of artistic visual stimuli (7). Also, esthetic interpretation and art production in general requires cognition, symbol processing, and memorization. In this setting, the prefrontal lobe acts as an executive center modulating symbol processing and cognition, but its activity is supervised by basal ganglia (8, 9). Neuroimaging studies, based on an esthetic-artistic perspective, have revealed simultaneous activation of both basal ganglia and (pre)frontal lobes during assessment of various visual stimuli (10–12). Magneto-encephalographic studies recording the activity of the frontal cortex during evaluation of abstract and classic art representations as well as visual assessment of photographs of landscapes, artifacts, and urban scenes have shown that this cortical region becomes “responsive” subsequent to visualization of “beautiful” art or photographs (12). Complex functional magnetic resonance imaging studies (fMRI) evaluating judgments of symmetry and esthetics have indicated activation of specific regions, such as bilateral ventral (pre)frontal cortex. However, when the subjects identified a certain pattern to be “beautiful” left intra-parietal sulcus, an area responsible for symmetry evaluation was also activated showing an increased signal on fMRI. The results suggest that symmetry perception was increased if in parallel the participants evaluated a beautiful object (10). Interestingly, transcranial magnetic stimulation studies have revealed that left (pre)frontal cortex is activated in parallel with right posteriors parietal cortex in “like–dislike” studies evaluating various paintings (13). All of these data support the fact that artistic creation is strongly linked to the functionality of the (pre)frontal lobe substantiating a neurobiological basis of neuroesthetics but at molecular level the chain of events is currently unknown.

### The Role of Parietal Lobe and Temporal–Parietal Junction in Artistic Creation

It is known that parietal cortex is specialized in coordinating eye movements, grasping, and other visual–motor actions. Moreover, this part of the cortex is activated by perception of object orientation, manual learning including the use various devices and machinery (14). At this stage, we can only speculate that it may very well relate to any type of manual art production as well. More importantly, in the realm of neuroesthetics, the activation of parietal cortex is associated with *positive* esthetic experiences. If healthy non-artistically trained subjects are requested to compare representational realist/figurative paintings or drawings and indeterminate/abstract art, fMRI records an increased activity in an area between the parietal and temporal lobes as well as in the temporal–parietal junction only after evaluating representational art. This suggests that specific content recognition in various paintings is determined by activation of this higher cortical region (15). Moreover, Kawabata and Zeki (7) have reported that the left temporal–parietal junction area is stimulated when the subjects describe “beautiful” visual art rather than neutral art.

Interestingly, magnetoencephalographic recordings of male and female parietal brain activity while evaluating artistic “beauty” have suggested different activation in men compared with women. In men, the activation was recorded in the right parietal cortex while in women the stimulation was equally represented in left and right parietal cortex. While the authors speculate that this significant difference might be related to human evolution, currently it is certain that men have a different activation than women when it comes to artistic evaluation. The activation of the right parietal lobe in men indicates that artistic “beauty” depends on global attention, and men use an exploratory strategy based on spatial coordinates only (16).

### The Role of Other Cortical Regions in Artistic Creation

**Figure 1:**
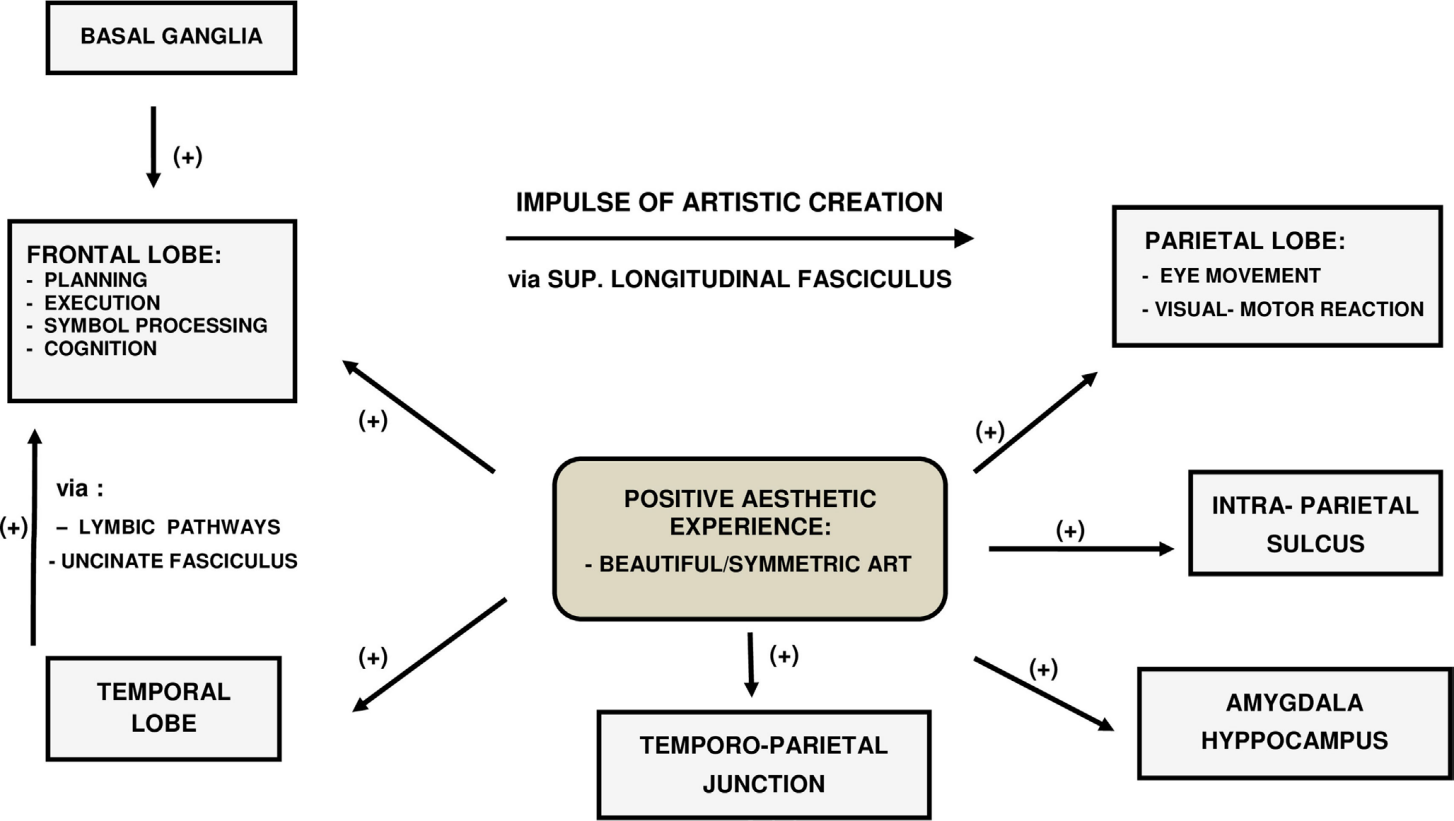
**The inter-relationship between the artistic creation, positive aesthetic experience and various CNS regions**.

Neuro-radiological research indicates that apart from (pre)frontal, parietal lobe, and temporal–parietal junction, other cerebral structures are also needed in art evaluation. Chakravarty (17) proposes a “neural circuitry of visual artistic production and appreciation” to include other cortical areas in addition to (pre)frontal, parietal, and temporal–parietal regions. Artistic creation and appraisal would include a “talk-back” between various part of the brain and also fine tuning of memory circuitry in the temporal cortex *via* the uncus. It is suggested that while “planning and execution” are modulated by various parts of the (pre)frontal lobe, “the actual “impulse” of creation is transferred from frontal cortex to the parietal cortex *via* superior longitudinal fasciculus. However, the (pre)frontal lobe functionality is influenced by stimuli received from temporal lobe *via* limbic pathways and uncinated fasciculus. In addition, artistic “beauty” signals are also processed in the amygdala and hippocampus. It is hypothesized that all these complex structures must be activated simultaneously in artistic evaluation as well as creation. Subsequently, once the image is conceptualized in the visual areas, the motor brain is activated for execution of that particular art. In fact, this hypothesis is complementary to Zeki’s “theory of multistage integration (TMI),” which states that visual brain is the most important integrative region modulating the functionality of brain in the context of neuroesthetics (17, 18). However, more studies are needed to confirm both Chakravarty’s and Zeki’s concepts, but the most important difficulty related to experimental work in this context is represented by standardization of clinical experiments. Nadal et al. (19) have highlighted that activation of certain parts of the brain related to neuroesthetics depends in an experimental setting not only on the subjects but also on the quality of the stimuli and of course on the actual experimental design. The current knowledge in this field revealed by the above mentioned functional studies including Zeki’s “TMI” and Charkravarty’s neural circuitry of visual artistic production and appreciation” is not sufficient to explain the relationships between cellular molecular changes described in brain parenchyma and artistic creation, but it represents a starting point for future research (Figure 1).

### Stroke and Artistic Creation

Cerebral stroke represents the final result of an ischemic or hemorrhagic event in a cerebral area. Depending on the clinical circumstances, stroke patients may survive and overcome their neurological deficit, which ensues after the acute episode. Experiments conducted in a murine model of stroke induced by middle cerebral artery occlusion have shown that cerebral necrosis is counteracted by neuroplasticity, which includes a process of neuronal regeneration starting very early after the acute event (20, 21). However, neuroplasticity requires poststroke angiogenesis to promote survival of newly formed neurons and neuronal steam cells (22). Remarkably, clinical improvements observed in some patients after stroke could be explained by neuroplasticity in the context of neurological recovery and compensation. In addition, after stroke, a visual artist may show active motor artistic skills related to artistic output with or without development of a totally different style compared with the prestroke period. We believe that in the poststroke period, active artistic skills re-appear as a result of motor recovery and/or compensation and are modulated directly by neuroplasticity.

Regarding the premordid style, we must highlight that all the artists discussed in this paper had previously acquired international fame and notoriety through their drawings and paintings created in a very specific individual style. The individual artistic style could be defined by certain traits and characteristics, which allows a potential recognition of the author. The theories explaining the style have evolved over time from Renaissance to modern times. While the Renaissance Alberti promotes a balanced realistic representation, modern authors, such as Martindale, suggest a psychological explanation of style, which claims that the artists is in a continuous quest for novel works in order to counteract the effects of habituation (23, 24). More importantly, for neurobiology of artistic creation, regardless the premorbid style, after a right hemispheric stroke, the new style is mainly characterized by a loss of correct spatial relationships, asymmetry determined by left-side neglect, use of elementary bold colors, simplification of images, as well as loss of both tri-dimensionality and “claire-obscure.” After a left hemispheric stroke, the new style is characterized by rigidity, loss of perspective and tri-dimensionality, and repetitiveness (25). However, these features have to be regarded with great caution as both right and left stroke artists are capable of circumventing these elements. In theory, any type of painting could be evaluated by Chatterjee’s assessment of art attributes. According to this method, a painting has perceptual attributes (balance, color saturation and temperature, depth, complexity, stroke) and representational attributes (abstraction, movement, emotion, realism, objective accuracy, and symbolism) (26).

## Methods and Results

The current manuscript is not focusing on the actual esthetic evaluation of the artistic attributes or style *per se*, but instead it gathers all the available relevant information related to any change in style after stroke which we term “poststroke style,” in patients with active artistic skills. The information related to poststroke style including any possible change was extracted from previously published reports. While the number of visual artists that presented with stroke is higher, we have selected only those cases with sufficient clinical information that have enabled us to make important clinical correlations related to poststroke artistic skills recovery and compensation.

In this context, our PubMed search (1995–2015) revealed 10 documented cases of internationally known artists that presented with right hemispheric stroke and poststroke active artistic skills (Table 1).

**Table 1:** **Artists with right hemispheric stroke**.

Patient’s name	Age	Clinical features	Neglect	Active artistic skills	Poststroke style	Reference
Anton Räderscheidt	75	Left hemiparesis Prosopagnosia	Present	Present	New style	Bäzner and Hennerici (29) Cantagallo and Della Sala (27)
Otto Dix	76	Left hemiparesis Decreased proprioception Apraxia Hemianopia	Present	Present	Not clearly established	Bäzner and Hennerici (29) Mazzucchi et al. (29) Cantagallo and Della Sala (27)
Johannes Thiel	70	LEFT hemiparesis including face decreased left hand motricity Additional fatal stroke	Absent	Present	Recovered premorbid style	Bäzner and Hennerici (28)
Federico Fellin	73	left hemiparesis Hemianopia Severe sensorimotor syndrome Dyslexia	Present	Present	Recovered premorbid style	Bäzner and Hennerici (28) Cantagallo and Della Sala (27)
Reynold Brown	59	Left hemiparesis Hemianopia	Present	Present	New style	Bäzner and Hennerici (28)
Tom Greenshields	75	Left hemiparesis Hemianopia Severe sensorimotor syndrome	Present	Present but as right handed artist had to learn to use left hand 8 years before stroke after an accident	New style	Bäzner and Hennerici (28) Cantagallo and Della Sala (27)
Guglielmo Lusignoli	67	Left hemiparesis	Present	present	Not indicated	Bäzner and Hennerici (28)
Lovis Corinth	53	Left hemiparesis Motor deficit Neuropsychological deficits	Present	Present	New style	Bäzner and Hennerici (28) Mazzucchi et al. (29)
Kurt Schwitters	57	Left hemiparesis	Present	Present	New style	Bäzner and Hennerici (28)
Segundo Agelvis	88	Left hemiparesis Hemianopia Apraxia Additional fatal stroke	Present	Present	New style	Mazzucchi et al. (29)

These patients were all males, and the average age of this selected group of patients was 69 years. All presented with left-hemiparesis and neglect. Active artistic skills recovery was recorded in all cases. In some cases, such as Räderscheidt, Fellini, and Corinth, the ability to draw was present within days after the stroke (27, 28). We could speculate that this was related to both poststroke recovery and compensation, but the proportion of each mechanism cannot be estimated. However, poststroke compensation could have been significant in the case of Reynold Brown, a famous American left handed visual artist who had to learn to use his right hand after his right cerebral stroke. As these artists lived in various countries, at various intervals of time, the investigational, therapeutic, and rehabilitation methods used in every case are different. In addition, the literature is not clear about the etiological type of stroke in every case. However, most of these artists presented with left hemineglect which interfered considerably with their poststroke style. Also, in the poststroke period, six of these artists adopted a new style while in the cases of Federico Fellini and Johannes Thiel, the regaining of the premorbid drawing and painting style was obvious (27, 28). Mazzucchi et al. (29) suggest the adoption of a new style in painters after right hemispheric stroke is characterized by the display of vivid colors, simplistic constructions, as well as loss of perspective and minute detail. In addition, these artists ignore the left visual field. These changes do not represent a less valuable artistic creation but on the contrary. For the spectator, the artistic message conveyed through the new style is very powerful. However, while we can associate the poststroke artistic activity with motor recovery and compensation, we do not know what determines and motivates the abrupt change of style after stroke.

### Clinical Cases of Artists with Right Hemispheric Stroke

**Figure 2:**
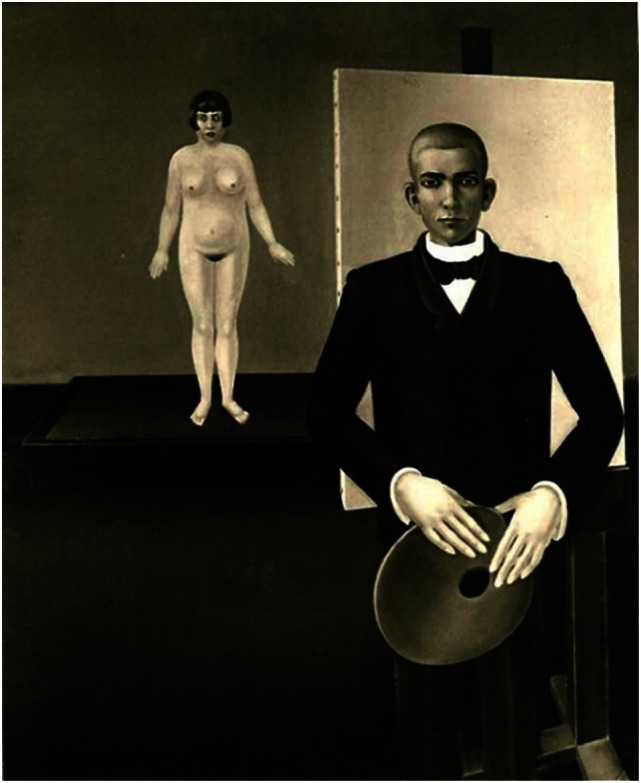
**Anton Räderscheidt: painter and model (pre-stroke)**.

- Perhaps the most thoroughly investigated case of stroke in an artist is represented by Federico Fellini, a renowned film director but also a cartoonist and visual artist. The artist presented with hemineglect after right parietal stroke, which did not affect his language or memory. A left visual field defect in the inferior quadrant suggested a lesion at the level of optic radiations. In addition, the patient had a sensory–motor deficiency on the left part of his body. He draw characters and human portrait sketches based on what he observed in his normal visual field omitting specific visual information from the left side. Interestingly, during the same period of time, he also produced drawings in which the unilateral neglect is very difficult to be identified. More intriguingly, Fellini did not show a writing deficit under any circumstances (dictation/spontaneously). It is reported that Fellini recovered completely his drawing abilities within 2 months after the stroke, but initially he started to draw within days after stroke. No style differences were noted between drawings created at 60 days after stroke and those produced before the ischemic event (27). Therefore, we may conclude that his artistic skills have “recovered” but at the present time, we do not have a clear understanding of his style recovery. The context becomes even more puzzling since Fellini’s perceptual and visual–motor neglect for left space were never corrected while both his skills and style recovered. Moreover, the above described recovery was associated with permanent neglect and motor deficit awareness.
- Johannes Thiel (1889–1962), a prolific graduate of Munchen and Stuttgart Art Academies, presented with a series of ischemic strokes in his early 70s. After the initial right cerebral stroke, he developed left facial paresis associated with left-side motor deficit but the patient recovered completely. The artist did not show any indication of neglect and as Fellini, he presented artistic skills recovery with similar premorbid style (30).
- After stroke other artists will regain their artistic skills but with subsequent development of a different style, often times more expressive. Lovis Corinth (1858–1925) is one of the most significant modern German painters of Expressionist attitude. In 1911, he presented with a right cerebral stroke and subsequently showed artistic skills recovery and a new style of expression. It is suggested that he had a left visual hemineglect and his paintings completed after his stroke area characterized by significant left visual field omissions, which may be explained by an attentional–spatial deficit. Although his characters display spatial deformities, the message of his art created after stroke is still impressive (31).
- One of the best documented cases associated with development of a new painting style is represented by Anton Raderscheidt (1892–1970), a significant member of the Dada current as well as a prolific Magischer Realismus adept from Koln, Germany (32). Anton Raderscheid developed a right cerebral stroke, which was associated with left hemiplegia and loss of the left visual field as well as left-side neglect. In addition, he showed prosopagnosia, which relates to difficulty in identifying familiar faces (32). Prior to his right hemispheric stroke, the artist represented icon-like characters with great accuracy and in great detail (Figure 2).

**Figure 3:**
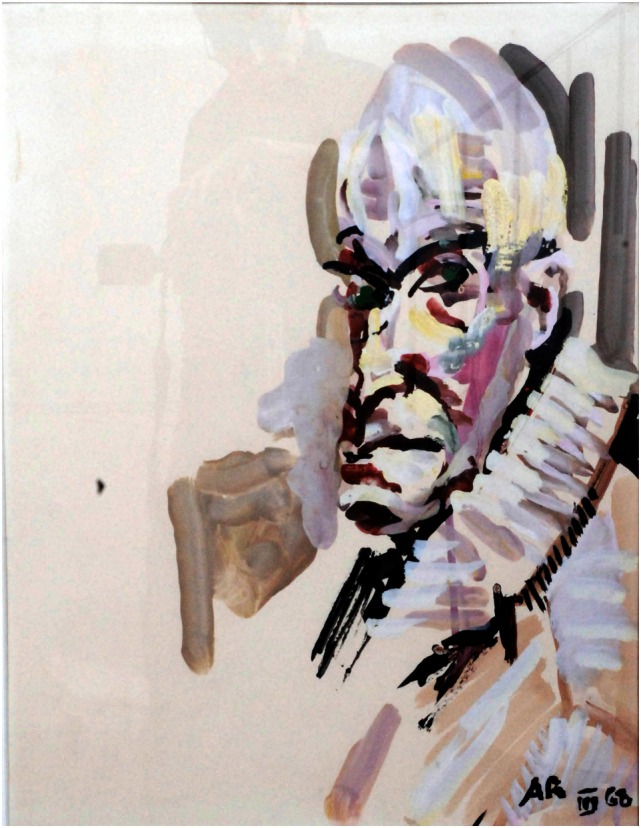
**Anton Räderscheidt: self-portrait 1 (post-stroke)**.

**Figure 4:**
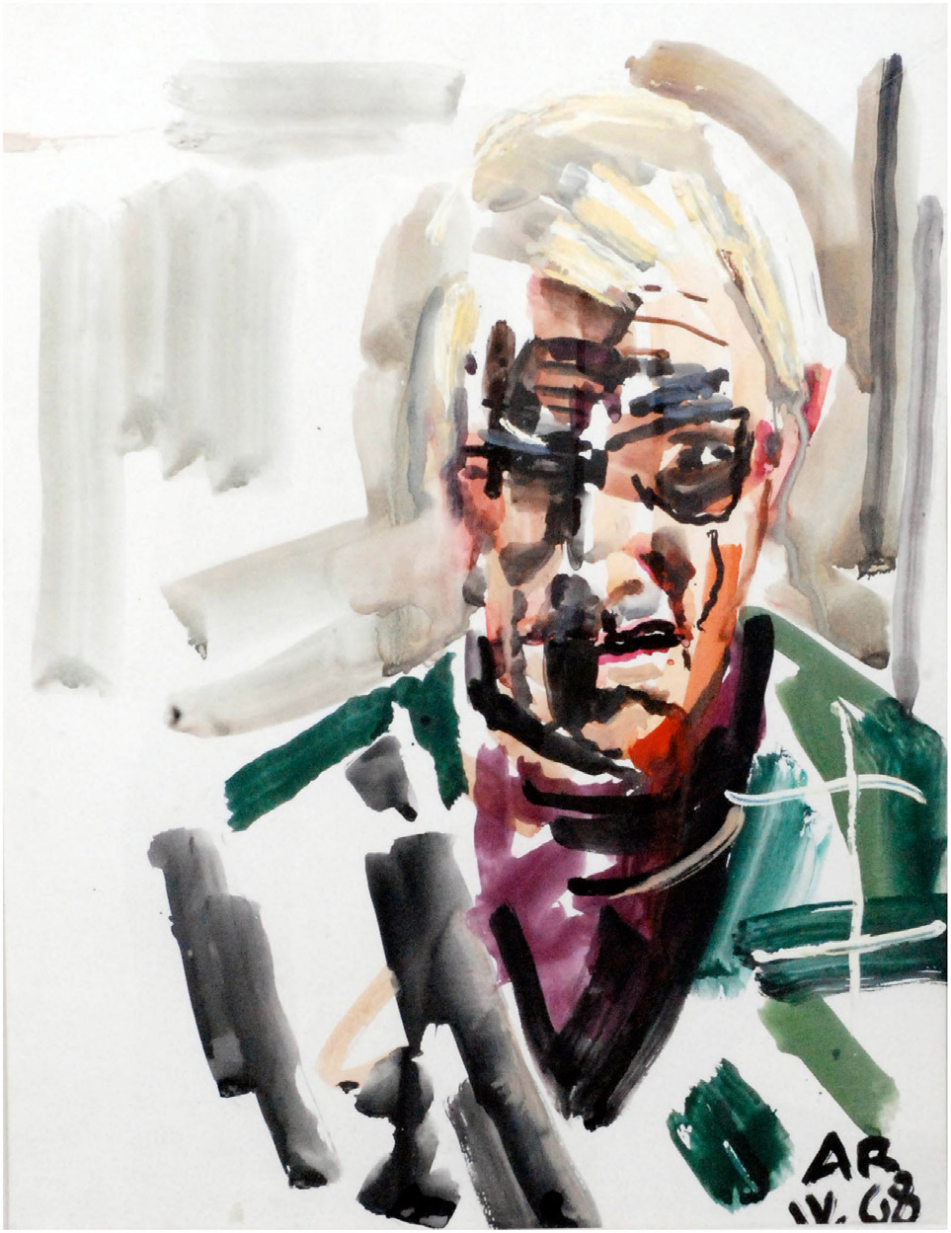
**Anton Räderscheidt: self-portrait 2 (post-stroke)**.

In the poststroke period, his portraits are constructed *only* on the basis of what he saw in his right visual field. However, his subjects showed a simple but more vibrant and eclectic image compared with the prestroke detailed portraits (Figures 3 and 4). In his autobiography, the artist mentions his interest in using vivid color in his poststroke paintings (31).

In other painters, the recovery of style is more problematic. Otto Dix (1891–1969), a Professor of Art at the Dresden Academy, was the promoter of large paintings depicting war scenes inspired by his experience as a soldier during the First World War. His compositions are notable for human caricature-like figures. Dix presented with right-side hemispheric stroke associated with left hemineglect and hemiparesis as well as left apraxia. Remarkably, at 4 days after the neurological event, he was capable to construct simple (trees) but with left visual field neglect. However, he appeared to compensate the neglect within 2 weeks. In this case, the artistic skills recovery was obvious but while some authors claim that he his style remained unchanged after the injury, others believe that his style has changed dramatically (28, 30).

Regarding the group of famous artists with left hemispheric stroke a PubMed search (1995–2015) revealed five cases (Table 2). There were four males and one woman in this selected group, and the average age of this selected group of subjects was 51 years. All presented with right-hemiparesis and aphasia. Artistic skills recovery was recorded in all cases, and motor compensation played a major role as well in all these cases. With a possible exception (Basaldella), all these artists adopted a new style after stroke. Apart from the extent of the lesion and neuroplasticity, the development of a new style after stroke may also be partly explained by the fact that all patients were prompted to work with a previously un-used left hand (28, 29). The style seen in painters after left hemispheric stroke is described by some authors as mechanical, employing light-flat colors and avoiding topics, which require perspective (29). Despite all these changes, these artists have been able to create impressive masterpieces, and in some instances, a clear qualitative evolution may be identified. As in the case of artists with right hemispheric stroke, these artists lived in various countries with different medical systems. There is limited published information related to their poststroke rehabilitation program and their etiological type of lesion. Therefore, we could make any correlation between severity, type of stroke, artistic skill recovery/compensation, and poststroke style. Also, while these artists presented with an active poststroke motor activity, we are agnostic about any potential postevent psychological or any factors that might have determined the adoption of a different style after stroke.

**Table 2:** **Artists with left hemispheric stroke**.

Patient’s name	Age	Clinical features	Neglect	Active artistic skills	Poststroke style	Reference
Katherine Sherwood	44	Right hemiparesis Aphasia Decreased right side motricity	Absent	Present	New style	Chatterjee et al. (33) Mazzucchi et al. (29)
Zlatio Boiyadjiev	48	Right hemiparesis Global aphasia Reduced verbal expression and comprehension Additional fatal stroke	Absent	Present	New style	Chatterjee et al. (33) Mazzucchi et al. (29)
Paul Gernez	52	Right hemiparesis Apraxia Aphasia Hemianopia Agraphia Anosmia Introversion	Absent	Present	New style	Boller et al. (25)
Gianfranco Fasce	54	Right hemiparesis Aphasia Additional fatal stroke	Absent	Present	New style	Mazzucchi et al. (29)
Afro Basaldella	59	Right hemiparesis Limited general motricity Dysarthria	Absent	Present	Recovered premorbid style	Mazzucchi et al. (29)

### Clinical Cases of Artists with Left Hemispheric Stroke

Katherine Sherwood and Zlatio (Zlatyiu) Boiyadjiev are two well-known modern artists who have suffered left hemispheric stroke and right hemiparesis. Both artists showed artistic skills recovery and subsequent to the acute event, they embraced new topics and styles, which could be described as energetic, fluid, and expressionist (29, 33). Clinical and artistic evolution observed in Katherine Sherwood and Zlatio Boiyadjiev suggests that both artists have learned to paint with the left hand as the right motor function was impaired after the stroke. It is suggested that the primary motor area (precentral gyrus) responsible for upper limb motor function is not involved in superior brain processing required for artistic creativity or esthetic interpretation. This reinforces the principle that a stroke affecting the dominant cerebral hemisphere including its primary motor area obliges the artist to re-learn drawing and painting using the healthy hand. Similar with the right hemispheric stroke, neuroplasticity has a paramount role in artistic skills recovery, but the major molecular players are yet to be identified.

**Figure 5:**
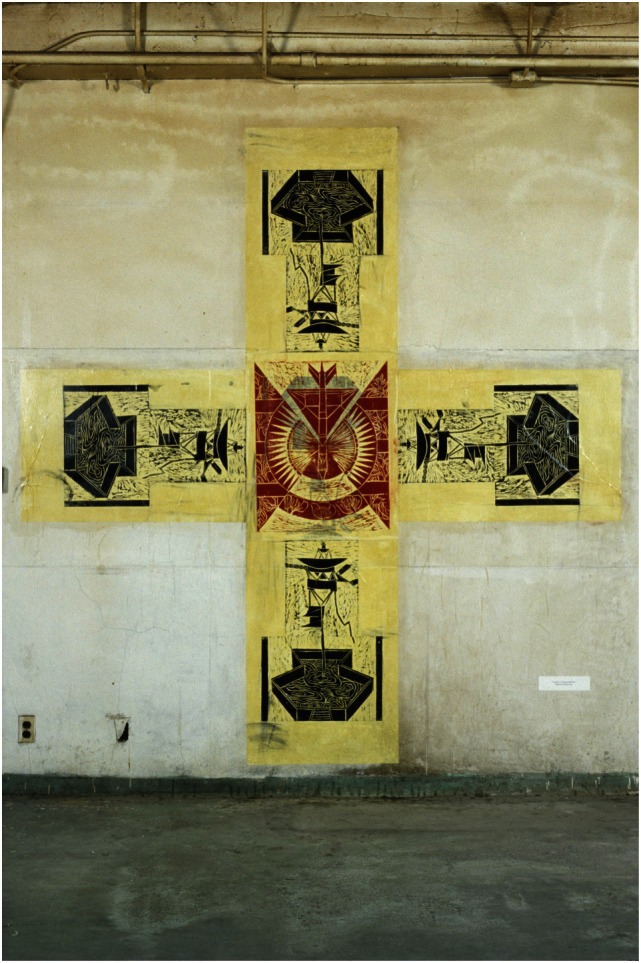
**Katherine Sherwood: Voyager’s Constant (pre-stroke)**.

**Figure 6:**
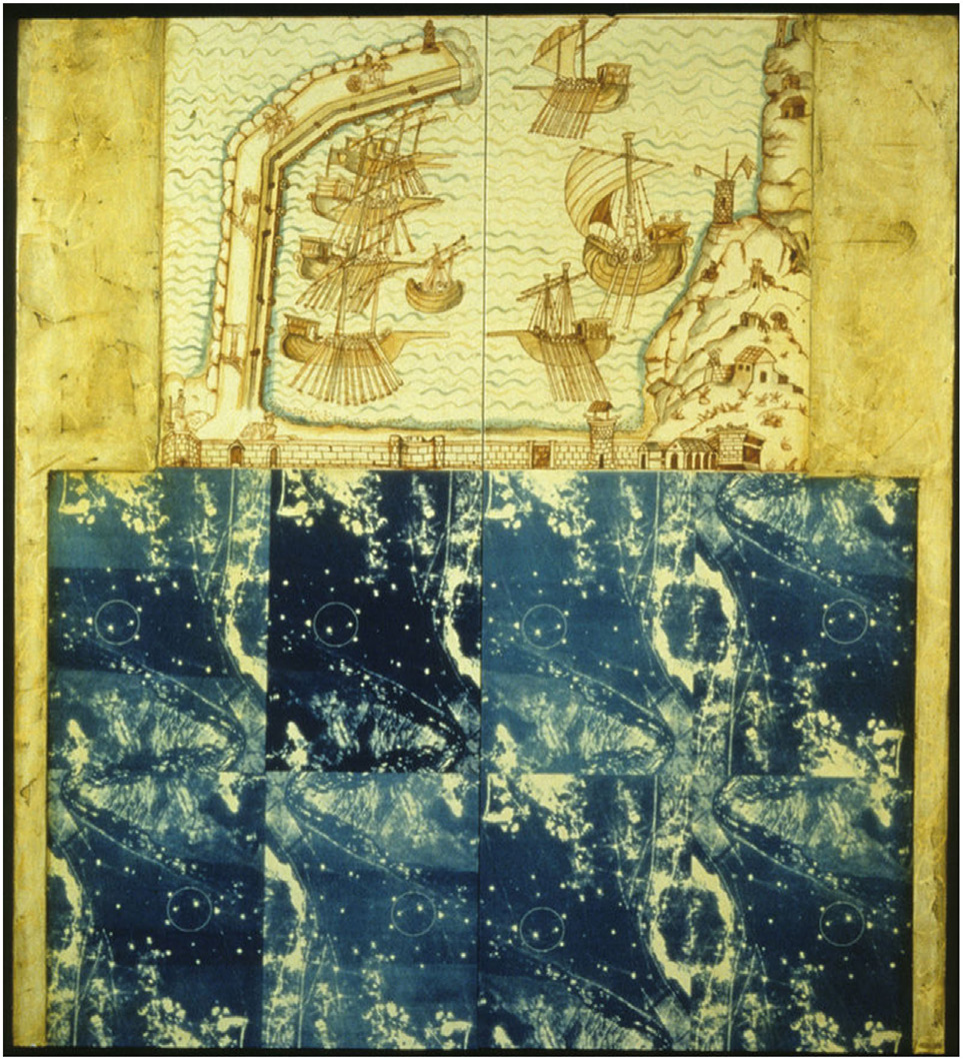
**Katherine Sherwood: test sites (pre-stroke)**.

**Figure 7:**
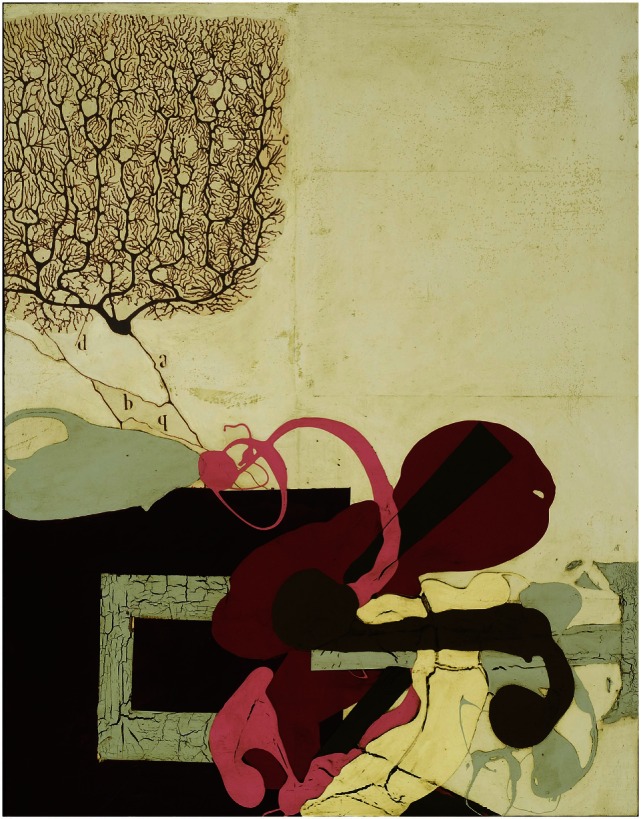
**Katherine Sherwood: Cajal’s Revenge (post-stroke)**.

Katherine Sherwood shows in her 1989 “Voyager’s Constant” (Figure 5) and in the “Test Sites” completed in 1992 (Figure 6) a clear focus on details and construction of intricate elements, while after stroke in her 2006 “Cajal’s Revenge” (Figure 7) displays new forms and elements suggesting a new evolving style.

Paul-Elie Gernez (1888–1948), a modern French artist trained at the Valencienne Academy, presented with left cerebral stroke at 52 years of age. He developed Wernicke type aphasia, hemianopia, and transient apraxia. His ability to speak was impaired but according to Alajouanine he maintained his premorbid painting style after stroke (25, 34). However, other authors suggest that he changed his style after stroke, his paintings been characterized by loss of spatiality and increased flatness as well as super-positioning of subjects (31). Interestingly, although he was aphasic, he begun to draw on the hospital bed within couple of days after stroke (31).

**Figure 8:**
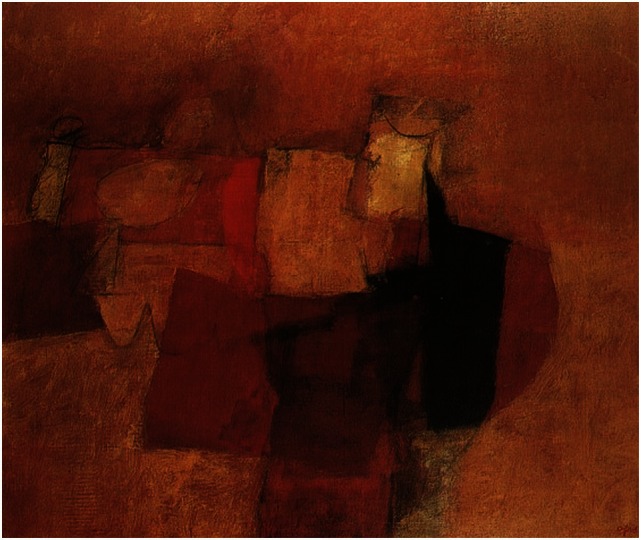
**Afro Basaldella: La Terza Baronessa**.

**Figure 9:**
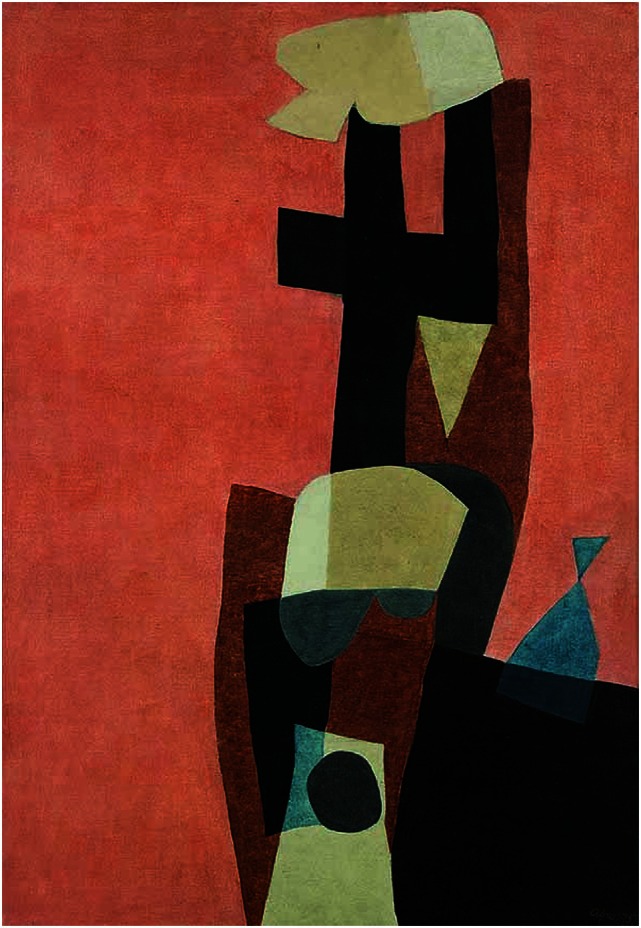
**Afro Basaldella: Tiresia**.

Afro Basaldella (1912–1976) is a famous representative of Scuola Romana and one of the most important artists associated with modernism and abstract art in Italy in the twentieth century who latter had an impressive career in the United States (29). Our documentation from the Afro Foundation Archive suggests that the artist suffered two left cerebral strokes in 1971. However, he recovered and continued to paint until a third left cerebral stroke took place in April 1975. Remarkably, Afro may represent a singular case of artist with left stroke who might have regained his style after recovering from stroke. There is not much difference in terms of construction and depth, color, and style between his 1970 “La Terza Baronessa” (Figure 8) and his poststroke 1975 “Tiresia” (Figure 9). Both are powerful masterpieces created with a similar technique and level of artistic skills.

A general evaluation of the poststroke work of the above mentioned artists with left stroke obviously contradicts the idea reiterated by Mazzucchi et al. (29) that after left cerebral stroke the style is rather mechanical. As suggested above, some of these artists show an impressive evolution.

### Poststroke Artistic Skills and Style

Numerous functional studies focusing on esthetic appraisals tests report a significant cerebral activation in specific cerebral regions such as the fronto-median cortex as well as the prefrontal cortex/inferior frontal gyrus, parietal lobe, and temporal–parietal region. As previously mentioned, Zeki and Chakravarty suggest even a system of activation pathways as well as a visual brain centered “multistage integration” system responsible for artistic output (17, 18). Furthermore, clinical cases of stroke described in artists have suggested a morphological basis of artistic creation. These studies have shown that esthetic judgment is associated with activation of well delineated cerebral areas highlighting the potential of high-level sensory centers as modulators of artistic neurobiology and neuroesthetics in general. In this context, processing of visual stimuli including evaluations as well as creation of visual art may require local cellular–molecular changes including new metabolic requirements. Jacobsen has been able to identify by fMRI an increase of blood oxygenation level-dependent (BOLD) in the region of prefrontal cortex in experiments evaluating the perception of beauty and symmetry (35). Subsequently, other groups have demonstrated that a prefrontal BOLD activation is followed by a cholinergic response, which could mediate the integration of visual stimuli with internal processing leading to certain action or behavior (36). It seems that this complex process is facilitated by the prefrontal alpha4beta2 nicotinic acetylcholine receptors (nAChRs) population, which mediate the transient glutamatergic–cholinergic interactions required for attention and cue evaluation (37). This indicates that these types of receptors could potentially play a significant role in the processes and reactions associated with esthetic evaluation and artistic creation. At the present time, the difficulty of investigating these receptors in dynamic life experiments precludes us from clarifying their role in artistic creation. We do not have enough experimental data to understand the molecular chain of events determined by ischemic or haemorrhagic destruction of brain parenchyma which could control artistic creation and/or evaluation. However, regarding the clinical outcome in patients with stroke, it is accepted that it this depends on the extent of the initial neurological deficits (38). Thus 80% of patients who survive the acute phase will present initially with functional deficits (e.g., paralysis, sensitivity disorders, aphasias, etc.), but some of these problems tend to subsequently subside or improve, at least in some patients. Young patients in good health and without other associated diseases will recover faster and better than elderly with co-morbidities. The neurological recovery and clinical improvement ultimately depend on the adult human brain potential for functional and anatomical re-organization (39, 40). Some authors consider that recovery relates not only to clinical improvements achieved *via* several mechanisms but also to a “restoration of function,” which was lost after neurological injury at cellular/molecular level (41). However, the mechanisms of neurological recovery are incompletely understood. Clinical studies have shown that up to 75% of stroke patients would present with upper arm functionality problems at several months after the injury, and the recovery at this level is more complex been achieved at a later stage than in the lower limb (42, 43). This could be partly explained by the therapeutic protocol, which concentrates more on the rehabilitation of lower limb than on the upper extremity as mobility of the patient is of paramount importance in preventing adverse effects of long-term immobilization (44). However, if we consider the motor activity required for drawing or painting, most of the artists discussed in this paper have shown an early recovery of their drawing skills. For example, both Fellini and Dix begun to draw within days after stroke and in most of the cases the recovery of their artistic drawing or painting abilities were complete within several months. We conclude that poststroke artistic abilities are not only the result of neurological recovery but also they are promoted by compensation. In the latter case, it seems that motor neural elements that have not been damaged by stroke would adapt and replace the motor components already injured by stroke (41). This is most obvious in the case of right handed artists, Katherine Sherwood and Zlatio Boiyadjiev who both suffered a left hemisphere stroke and subsequently had to learn to draw with left hand developing new artistic styles, considered by some art critics more energetic and “more visceral” than the premorbid style (33). Numerous clinical studies conducted in patients after stroke have indicated that compensation is associated with activation of cerebral areas which normally are not responsible of performing certain motor tasks (45, 46). We can only assume that this principle applies also to the cases mentioned in this manuscript.

**Figure 10:**
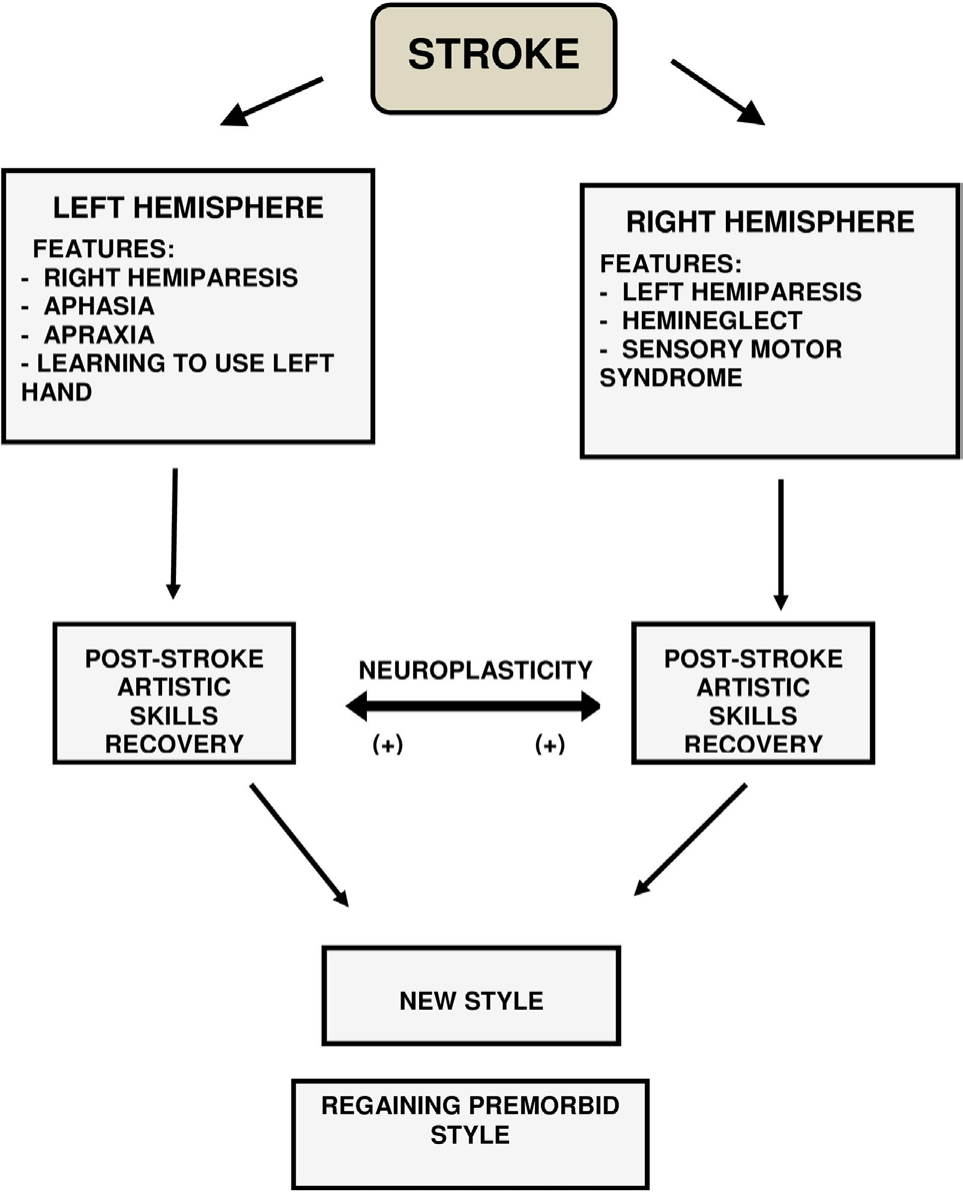
**Stroke, neuroplasticity, style and post-stroke artistic skills recovery**.

Regarding the neurological recovery and compensation and the level of the actual motor performance, the previously published reports fail to provide sufficient information. Therefore, we do not know if the motor artistic production was performed *technically* as before the stroke injury or it was completed in a new fashion with the introduction of new alternative patterns or even different effectors. However, artistic motor skills recovery and compensation are intrinsically linked to neuroplasticity of the brain. By definition, neuroplasticity allows the neural tissue to “adapt” itself subsequent to various factors including tissue destruction. This “adaptation” includes re-organization of various neural components in terms of function and connection at molecular and system levels as well as at the functional level (45). The ultimate result is represented by re-tuning of various morphological–functional and cellular structures (Figure 10).

Regarding the odds for artistic skills recovery and compensation with or without regaining the prestroke style, this may well depend on factors that modulate clinical recovery in the any stroke patient such as age, previous ischemic events, and aphasia (47).

We could argue that artistic skills recovery with or without “restitutio ad integrum” of the style may be explained not only by neuroplasticity but also by limited destruction of certain regions of the brain responsible for planning, creation, and execution of the artistic work.

As the style depends not only on the integrity of motor centers and pathways but also on the functionality of other superior centers related to psychological functions including cognition, memory, semantics, as well as centers that coordinate the eye function and perception and orietation in space, we could speculate that modulation of poststroke style is complex.

In general, the majority of patients with stroke would develop ischemia in frontal, temporal–parietal, or occipital cerebral areas. As previously mentioned, functional and experimental studies have revealed that in these areas are located specific centers that may modulate the artistic processing and interpretation (7–10, 12, 14). An ischemic lesion in the medial orbito-frontal lobe would theoretically impair evaluation of any artistic stimuli while a lesion in the basal ganglia would make cognition and symbol processing very difficult or impossible.

It is also possible that destruction of a central nervous system region would subsequently determine disinhibition of certain centers that would promote a certain psychological behavior and ultimately artistic style but all these scenarios are yet to be validated in clinical studies.

However, in both right and left cerebral strokes, we can assume that neuroplasticity has a primordial role in artistic rehabilitation. In these cases, extrapolating the principles derived from the study of non-artistic neurological patients with long-term persistent motor-sensitive deficits to an artist with stroke, suggests that neuroplasticity is not only responsible for neurological recuperation but also promotes the development of a new poststroke artistic style. It is already known and widely accepted by the scientific community that patients with long-term neurological issues develop new compensatory adaptive mechanisms. Therefore, poststroke active artistic skills with subsequent development of a new style could represent a new adaptative reaction prompted by cerebral damage. Interestingly, some authors have suggested that neglect in the acute stage after stroke is a negative predictor of functional recovery (48). However, in cases of artists with right hemispheric stroke, poststroke neglect explains the construction and design of drawings and paintings. At least on a limited term, this is not associated with impairment of poststroke artistic skills. More importantly, the findings revealed by clinical studies conducted in artists after stroke could be extrapolated to a selected group of non-artistically trained neurological or psychiatry patients when evaluating in general their status. The artistic skills recovery and compensation with development of a new style or regaining of the previous prestroke style could represent a significant clinical element which must be further assessed in future standardized medical studies. It hoped that these types of standardized clinical studies will reveal important information related not only to neurobiology of artistic creation but also to neurorehabilitation after stroke. However, any extrapolation of results related to artistic skills recovery and compensation between artists and non-artists has to be conducted with caution as the effect of cerebral lesions is different in these two groups of patients due to expanded cortical representation in artists, which is secondary to lifelong formal training (25).

## Author Contributions

EP and RM have significantly contributed to design, data collection and evaluation as well as writing-up the manuscript. KS has contributed to design, data evaluation and graphics. AP-W, AB, LA have contributed to data selection and interpretation.

## Conflict of Interest Statement

The authors declare that they have no financial, commercial or other relationships that might be perceived by the academic community as representing a potential conflict of interest by publication of this manuscript.
